# HIV-1 transmission networks across Cyprus (2010-2012)

**DOI:** 10.1371/journal.pone.0195660

**Published:** 2018-04-23

**Authors:** Leondios G. Kostrikis, Johana Hezka, Dora C. Stylianou, Evangelia Kostaki, Maria Andreou, Ioanna Kousiappa, Dimitrios Paraskevis, Ioannis Demetriades

**Affiliations:** 1 Department of Biological Sciences, University of Cyprus, Aglantzia, Nicosia, Cyprus; 2 Department of Hygiene, Epidemiology and Medical Statistics, Medical School, National and Kapodistrian University of Athens, Goudi, Athens, Greece; 3 AIDS Clinic, Larnaca General Hospital, Larnaca, Cyprus; Fudan University, CHINA

## Abstract

A molecular epidemiology study of HIV-1 infection was conducted in one hundred diagnosed and untreated HIV-1-infected patients in Cyprus between 2010 and 2012, representing 65.4% of all the reported HIV-1 infections in Cyprus in this three-year period, using a previously defined enrolment strategy. Eighty-two patients were newly diagnosed (genotypic drug resistance testing within six months from diagnosis), and eighteen patients were HIV-1 diagnosed for a longer period or the diagnosis date was unknown. Phylogenetic trees of the *pol* sequences obtained in this study with reference sequences indicated that subtypes B and A1 were the most common subtypes present and accounted for 41.0 and 19.0% respectively, followed by subtype C (7.0%), F1 (8.0%), CRF02_AG (4.0%), A2 (2.0%), other circulating recombinant forms (CRFs) (7.0%) and unknown recombinant forms (URFs) (12%). Most of the newly-diagnosed study subjects were Cypriots (63%), males (78%) with median age 39 (Interquartile Range, IQR 33–48) reporting having sex with other men (MSM) (51%). A high rate of clustered transmission of subtype B drug-sensitive strains to reverse transcriptase and protease inhibitors was observed among MSM, twenty-eight out of forty-one MSM study subjects (68.0%) infected were implicated in five transmission clusters, two of which are sub-subtype A1 and three of which are subtype B strains. The two largest MSM subtype B clusters included nine and eight Cypriot men, respectively, living in all major cities in Cyprus. There were only three newly diagnosed patients with transmitted drug resistant HIV-1 strains, one study subject from the United Kingdom infected with subtype B strain and one from Romania with sub-subtype A2 strain, both with PI drug resistance mutation M46L and one from Greece with sub-subtype A1 with non-nucleoside reverse transcriptase inhibitors (NNRTI) drug resistance mutation K103N.

## Introduction

In the last twenty years, combined antiretroviral drug therapy (cART), has been developed to specifically target HIV-1 with outstanding success, resulting in a dramatic decrease in mortality among HIV-1-infected individuals. However, the genetic variability of HIV-1 constitutes the most striking challenge in effectively treating HIV-1 infection. Specifically, the accumulation of drug resistant mutations during suboptimal therapy severely affects the clinical benefits of cART, leading to impaired therapy outcome [1–3] and the transmission of drug-resistant HIV-1 strains to newly-infected individuals in European countries [4–8], recently reported at just below 9% among newly-diagnosed individuals from 26 European countries between 2008 and 2009 [5]. Furthermore, according to the most recent molecular epidemiology study of HIV-1 infection in Europe, the most prevalent Group-M subtypes and inter-subtype circulating recombinant forms (CRFs) were subtype B (66.1%), followed by sub-subtype A1 (6.9%), subtype C (6.8%) and CRF02_AG (4.7%) with significant variances in subtype distribution among European countries, immigrant populations and patient risk-groups [9].

The first molecular epidemiological study for the HIV-1 infection in Cyprus, constituting the eastern European Union frontier in the Mediterranean Sea, was reported in 1995 [10]. HIV-1 was initially reported in Cyprus in the mid-1980s and the first reported HIV-1-infected patient in Cyprus was a young woman who reported living in the United States who was diagnosed in 1986 and died in 1987 [10]. Subsequently, the HIV-1 infection in Cyprus has been studied by densely sampled prospective molecular epidemiological studies of newly diagnosed patients (88% registered HIV-1-infected individuals until 2009) [11–13]. The main findings from the aforementioned HIV-1 molecular epidemiological studies in Cyprus is first, the high genetic heterogeneity of HIV-1 infection in the island as a result of a continuous influx of new HIV-1 strains from many countries, mainly from African countries, and second, the low transmitted resistance to HIV-1 antiretroviral drugs.

As part of our ongoing effort to monitor the genetic diversity of HIV-1 infection and the transmission of antiretroviral drug resistant HIV-1 strains in Cyprus, in this molecular epidemiological study we generated and analyzed HIV-1 *pol* sequences from one hundred HIV-1 diagnosed and untreated patients in Cyprus between 2010 and 2012 (65.4% of reported HIV-1 infections in Cyprus in this three-year period), using a previously defined enrolment strategy and previously established experimental procedures [11–13]. Furthermore, we examined the reported risk factors and other epidemiological information in an effort to gain further understanding into risks underlying the observed HIV-1 transmission networks in Cyprus during the three-year period, between 2010 and 2012.

## Material and methods

### Study subjects

For the period 2010 to 2012 blood samples were obtained from one hundred consenting HIV-1-infected individuals from the AIDS Clinic of Larnaca National Hospital, representing 65.4% of all the reported HIV-1 infections in Cyprus (area controlled by the Republic of Cyprus) in this three-year period. The blood samples from these individuals had been taken for standard genotypic drug resistance diagnostic purposes between January 2010 and September 2012 and were retrospectively added to this study after written consent from the study subjects as previously described [11–13]. Specifically, an informed consent form was signed by each subject and a questionnaire containing clinical, demographic and epidemiological information was filled by qualified medical personnel at the AIDS Clinic of Larnaca National Hospital. All samples and questionnaires were coded with a laboratory identifier number so as not to reveal the personal identity or the hospital registration identities of the study subjects. All blood samples were processed at the Laboratory of Biotechnology and Molecular Virology of the University of Cyprus within the same day of sampling. The majority of study subjects were Greek-Cypriots, although a number reported traveling or living abroad in the past. The HIV-1 sero-diagnosis of each subject was previously established by commercial enzyme-linked immunoassay and confirmed by Western blotting. All blood samples were processed at the Laboratory of Biotechnology and Molecular Virology of the University of Cyprus on the same day of sampling. This study is in conformity with regulations by the National Bioethics Committee and by the National Data Protection Commission in Cyprus.

### PCR amplification of pol region (protease and partial reverse transcriptase) and determination of drug-resistant mutations

HIV-1 sequences encoding approximately 1461 nucleotides of the *pol* (protease, PR and partial reverse transcriptase, RT) region were amplified from each sample by reverse transcription nested PCR (RT-PCR) using plasma HIV-1 RNA. The detailed experimental method for the amplification of the *pol* region by RT-PCR using plasma HIV-1 RNA was described by Kousiappa *et*. *al*, [13]. As part of our previously established methodology, in each sample, the DNA sequences encoding the *pol* regions were determined by population sequencing using the second-round amplified PCR product as the template and two sequencing primers (2136 and 3462) [13]. DNA sequence reactions were performed by the BigDye Terminator Cycle Sequencing kit and sequenced with the ABI 3130 genetic analyzer (Applied Biosystems, Foster City, CA) according to the manufacturer’s recommendations. HIV-1 drug resistance interpretation was defined based on the World Health Organization’s (WHO’s) list of drug resistance mutations for surveillance purposes, which was updated on 2009 [14]. Assessment of the levels of expected resistance to each of the three classes (nucleoside reverse transcriptase inhibitors, NRTI; non-nucleoside reverse transcriptase inhibitors, NNRTI; and protease inhibitors, PI) on the therapeutic response was predicted using the Stanford genotypic resistance interpretation algorithm (HIVdb version 8.3) [15] using the International AIDS Society-USA (IAS-USA) drug resistance mutation list, which was last updated on 2015 [16].

### Phylogenetic analysis

HIV-1 subtyping was performed using the REGA HIV-1 & 2 automated subtyping tool (Version 2.0) [17, 18] and confirmed by phylogenetic analysis using a global set of sequences (N = 216) representative of all pure HIV-1 subtypes and the most prevalent CRFs as references. Reference **sequences** were obtained from the Los Alamos database at Los Alamos National Laboratory (online), http://www.hiv.lanl.gov (last updated on 2017). Phylogenetic trees were reconstructed using approximately maximum likelihood (ML) method as implemented in the FastTree (version2.1) program [19] using the generalized time-reversible (GTR) model of nucleotide evolution with the “CAT” approximation for different evolutionary rates across sites [19]. To explore putative recombinant patterns in the viral sequences, we performed a bootscanning analysis using SimPlot (version3.5.1) [20]. Putative recombinants were confirmed by phylogenetic analysis using partial fragments showing discordant phylogenetic signals against a reference set of HIV-1 group pure subtypes obtained from the Los Alamos database. Bootscanning was performed with a sliding window of 400 nucleotides overlapped by 50 nucleotides to define the recombinant assembly. Details about identification of the phylogenetic clusters have been described previously [21]. In brief, phylogenetic clusters were defined as those with bootstrap support greater than 85% (phylogenetic confidence criterion), and with a mean genetic distance of fewer than 0.015 nucleotide substitutions per site.

## Results

Eighty-two study subjects were newly diagnosed and blood was drawn within one month of HIV-1 diagnosis. The remaining eighteen patients were either chronically-infected (seven patients were diagnosed before 2009 and three in the three-year period 2010 to 2012) or with unknown date of HIV-1 diagnosis (eight patients). Study subjects were retrospectively enrolled in this study after written consent followed by the submission of a questionnaire containing clinical, demographic and epidemiological information that was filled by qualified medical personnel at the AIDS Clinic of Larnaca National Hospital. Patients who did not wish to participate were not asked to provide a reason for choosing to do so. The staff who completed the questionnaire did not report specific patient group(s) (i.e. gender, sexual orientation, IV drug use etc.) who were unwilling to participate in the study. A detailed description of the clinical profile of each patient is presented in Table 1. Epidemiological, demographical and clinical data were collected from each consenting study subject as previously described [5, 7, 11–13].

**Table 1:** Clinical and epidemiological information for study patients.

*Patient* ^*a*^	*Sex* ^*b*^	*Age (years)* ^*c*^	*Collection date* ^*d*^	*Last negative test date* ^*e*^	*Positive test date* ^*f*^	*Country of origin* ^*g*^	*Transmission risk group* ^*h*^	*CD4 (cells/mm*^*3*^*)*	*HIV-1 RNA* *(copies x 10*^*4*^*/ml)*	*Epidemiological information* ^*i*^
CY269	M	45	01/10	03/08	12/09	Cyprus	HC	553	2.71	Cypriot citizen. Infected in Greece by unprotected sex with a sex worker.
CY270	M	67	01/10	06/05	06/05	Cyprus	HC	355	1.25	Cypriot citizen. Born in Cyprus and lived in the U.K. Diagnosed HIV-1 seropositive in the U.K.
CY271	F	23	01/10	06/08	12/09	Cameroon	HC	912	0.54	Cameroon citizen. Arrived in Cyprus as a political refugee. Infected either in Cameroon or Cyprus. Last negative HIV-1 test was done in Cameroon.
CY272	F	28	01/10	03/09	12/09	Cameroon	HC, OHPC	401	0.65	Cameroon citizen. Arrived in Cyprus as political refugee. Infected either in Cameroon or Cyprus. Last negative HIV-1 test was done in Cameroon.
CY273	M	32	02/10	06/09	01/10	Cyprus	HBC	119	3.80	Cypriot citizen. Infected in Cyprus.
CY274	M	53	02/10	05/02	02/10	U.K.	HC/Tattoo	N/A	1.85	U.K. citizen. Infected in the Netherlands or U.K. He reported that the likely route of HIV-1 infection was a tattoo. Genetic analysis of isolated HIV-1 strains revealed a drug resistance mutation (M46L) for PI.
CY275	M	25	04/10	NP	02/08	Cyprus	HBC	N/A	0.32	Cypriot citizen. Infected in Greece. Found HIV-1 positive in Greece. He reported that the likely route of HIV-1 infection was unprotected sex.
CY276	M	22	04/10	05/09	03/10	Cyprus	HBC	N/A	2.69	Cypriot citizen. Infected in Cyprus. Diagnosed HIV-1 positive prior to a surgery. Homosexual partner of study subject CY277. HIV-1 strain isolated from the patient is in a large phylogenetic cluster with eight other patients including study subject CY277 (See Fig 1B, Cluster 10)
CY277	M	17	04/10	NP	03/10	Cyprus	HBC	N/A	23.30	Cypriot citizen. Infected in Cyprus; Homosexual partner of study subject CY276 HIV-1 strain isolated from the patient is in a large phylogenetic cluster with eight other patients including study subject CY276 (See Fig 1B, Cluster 10).
CY278	M	50	04/10	-/85	03/10	Cyprus	HBC	N/A	2.39	Cypriot citizen. Infected in Syria. Reported having sex with men HIV-1 strain isolated from the patient is in a large phylogenetic cluster with eight other patients (See Fig 1B, Cluster 10).
CY281	F	55	05/10	NP	05/10	Cyprus	HC	N/A	3.16	Cypriot citizen. Most likely, she was infected in Greece by her bisexual ex-husband, who lives in Greece.
CY282	M	50	05/10	NP	U	Cyprus	HBC	482	2.40	Cypriot citizen. Infected in Thailand or Russia. Had an unprotected sexual contact with a transsexual sex worker. HIV-1 strain isolated from the patient is phylogenetically related with three patients (See Fig 1B, Cluster 7)
CY285	M	25	07/10	06/05	06/10	Romania	HC	N/A	0.05	Romanian citizen. When he arrived in Cyprus he was tested HIV-1 seronegative. Diagnosed HIV-1 positive during hospitalization.
CY286	M	25	07/10	MD	06/10	Cyprus	HBC	774	68.70	Cypriot citizen. Hospitalized in Nicosia with fever and Lymphadenopathy. Started HIV-1 cART in 2010. HIV-1 strain isolated from the patient is phylogenetically related with four patients (See Fig 1B, Cluster 7)
CY287	M	28	07/10	06/05	07/10	Cyprus	HBC	445	1.23	Cypriot citizen. Hospitalized in Nicosia hospital with fever, headache and diarrhea. HIV-1 strain isolated from the patient is in a phylogenetic cluster with two other patients (See Fig 1B, Cluster 6).
CY288	M	36	08/10	N/A	N/A	Cyprus	N/A	592	5.66	N/A
CY289	F	22	08/10	N/A	N/A	Romania	U	43	39.20	Romanian citizen. Infected in Romania and received HIV-1 cART therapy in Romania. She died in 2010 after hospitalization with pneumonia.
CY290	M	25	08/10	09/09	08/10	Cyprus	HBC	667	7.24	Cypriot citizen. Diagnosed HIV-1 positive during routine blood analysis. HIV-1 strain isolated from the patient is in a large phylogenetic cluster with eight other patients (See Fig 1B, Cluster 10)
CY291	M	38	08/10	MD	11/09	Cyprus	HBC	29	4.54	Found HIV-1 positive in 2009 during hospitalization with jaundice. In 2010, he was hospitalized due to recurrent pneumonia and high fever.
CY292	F	26	09/10	06/05	09/10	Lithuania	HC	223	3.52	Lithuanian citizen. She was HIV-1 negative when she immigrated to Cyprus. Her husband is HIV-1 negative. Diagnosed HIV-1 positive in the first trimester of pregnancy.
CY293	M	36	09/10	06/05	06/05	Cyprus	HBC	612	5.18	Cypriot citizen. Infected in Greece. He reported having sex with men.
CY294	M	27	09/10	03/10	09/10	Cyprus	HBC	538	0.703	Cypriot citizen. Diagnosed HIV-1 positive during pre-surgery procedures. HIV-1 strain isolated from the patient is in a phylogenetic cluster with study subject CY320 (See Fig 1B, Cluster 8)
CY295	M	31	10/10	N/A	10/10	Cyprus	HBC	710	1.28	Cypriot citizen. Infected in Cyprus. Homosexual partner of study subject CY296. HIV-1 strain isolated from the patient is in a phylogenetic cluster with three other study subjects including CY296 (See Fig 1B, Cluster 5)
CY296	M	18	10/10	01/10	10/10	Cyprus	HBC	396	1.70	Cypriot citizen. Infected in Cyprus. Homosexual partner of study subject CY295. HIV-1 strain isolated from the patient is in a phylogenetic cluster with three other study subjects including CY295 (See Fig 1B, Cluster 5)
CY299	M	39	11/10	06/05	11/10	Cameroon	OHPC	7	3.55	Cameroon citizen. Infected either in Cameroon or in Cyprus.
CY301	F	39	12/10	07/05	11/10	Romania	U	16	29.00	Romanian citizen. HIV-1 testing during immigration procedures was negative. Diagnosed HIV-1 positive during a pre-surgery procedure.
CY302	M	24	12/10	06/05	12/10	Romania	HBC	81	34.30	Romanian citizen. Infected in Cyprus. HIV-1 testing during immigration procedures in 2007 was negative.
CY305	M	33	01/11	N/A	12/10	Cyprus	HBC	14	2.62	Cypriot citizen. Presented with Kaposis sarcoma. On HIV-1 CART since 2011.
CY307	M	31	02/11	07/05	12/10	Togo	HC/OHPC	159	2.17	Citizen of Togo. HIV-1 testing during immigration procedures in 2009 was positive.
CY308	M	36	02/11	07/05	02/11	Cyprus	HC	4	6.49	Cypriot citizen. Diagnosed HIV-1 positive during routine check-up.
CY310	M	40	02/11	N/A	02/11	Cyprus	HC	35	8.11	Cypriot citizen. Diagnosed HIV-1 positive during hospitalization with pancytopenia and bleeding gums. He reported that HIV-1 infection was likely contracted in Greece. HIV-1 strain isolated from the patient is in a phylogenetic cluster with study subject CY331 (See Fig 1B, Cluster 9)
CY311	F	43	02/11	N/A	02/11	Cyprus	U	340	4.54	Cypriot citizen. Diagnosed HIV-1 positive during routine check-up. Her husband is HIV-1 negative.
CY313	M	48	03/11	09/10	15–211	Cyprus	HBC	465	1.67	Cypriot citizen. Diagnosed HIV-1 positive during standard blood donation testing procedures.
CY314	F	47	03/11	N/A	03/11	Romania	U	285	2.17	Romanian citizen. Diagnosed HIV-1 during a routine check-up during hospitalization.
CY315	M	37	03/11	06/05	03/11	Romania	HC	59	398.00	Romanian citizen. Infected in Romania. Diagnosed with HIV-1 when hospitalized with tuberculosis. He lives in Cyprus since 2010. Married to study subject CY319. He reported that HIV-1 infection was likely contracted in Romania.
CY316	F	70	03/11	N/A	03/11	U.K.	U	31	166.00	U.K. citizen. Diagnosed HIV-1 positive during pre-surgery procedure for lymphoma enterectomy.
CY317	M	23	04/11	N/A	02/11	Cyprus	HBC	814	0.78	Cypriot citizen. Infected in Cyprus.
CY318	F	36	04/11	06/05	N/A	Ukraine	U	320	1.56	Ukrainian citizen. Immigrated from Ukraine and lives in Cyprus. Infected in Cyprus. Diagnosed HIV-1 positive during an immigration visa renewal procedure. Husband is HIV-1 negative.
CY319	F	34	04/11	N/A	03/11	Romania	HC	32	176.00	Romanian citizen. Infected in Romania. Her husband is study subject CY315.
CY320	M	28	04/11	07/05	02/11	Romania	U	400	0.24	Romanian citizen. Diagnosed HIV-1 positive during a standard check-up prior to incarceration. HIV-1 strain isolated from the patient is in a phylogenetic cluster with study subject CY294 (See Fig 1B, Cluster 8)
CY321	M	32	04/11	N/A	01/11	Occupied Cyprus	HBC	360	7.84	Cypriot Citizen (Turkish Cypriot). Diagnosed HIV-1 seropositive on a routine check-up.
CY322	M	18	04/11	N/A	03/11	Cyprus	HBC	509	4.45	Cypriot Citizen. HIV-1 strain isolated from the patient is in a phylogenetic cluster with three other study subjects (See Fig 1B, Cluster 5)
CY323	F	23	04/11	N/A	06/05	Romania	Vaccination	9	4.02	Romanian citizen. She was infected in Romania and reported that the likely route of HIV-1 infection was a vaccination procedure in Romania. She also reported receiving HIV-1 antiviral treatment between 2003 and 2008 in Romania.
CY324	M	39	04/11	07/08	03/11	Greece	HC	441	22.60	Greek citizen. Resides in Cyprus since 2001. Diagnosed HIV-1positive when presented with genital herpes. Heterosexual partner of study subject CY325. HIV-1 strain isolated from the patient is in a phylogenetic cluster with study subject CY325 (See Fig 1B, Cluster 4)
CY325	F	28	04/11	12/07	03/11	Romania	HC	1151	0.28	Romanian citizen. Infected in Cyprus. Resides in Cyprus since 2007. Heterosexual partner of study subject CY324. HIV-1 strain isolated from the patient is in a phylogenetic cluster with study subject CY324 (See Fig 1B, Cluster 4)
CY328	F	29	05/11	06/10	03/11	Mali	HC	319	14.70	Mali citizen. Infected in Cyprus. Diagnosed HIV-1 positive during examination prior to surgery.
CY329	M	29	05/11	N/A	N/A	Cyprus	U	649	18.70	Cypriot Citizen. HIV-1 strain isolated from the patient is in a phylogenetic cluster with study subject CY363 (See Fig 1B, Cluster 2)
CY331	M	45	06/11	06/05	05/11	Cyprus	HC	667	1.56	Infected in Cyprus. Diagnosed HIV-1 positive during a routine check-up. He reported having extramarital affairs. His wife is HIV-1 negative. HIV-1 strain isolated from the patient is in a phylogenetic cluster with study subject CY310 (See Fig 1B, Cluster 9)
CY332	M	25	06/11	01/11	06/11	Cyprus	HBC	193	1.48	Cypriot citizen. Infected in Cyprus. Imprisoned at time of HIV-1 diagnosis. Reported having sex with men. HIV-1 strain isolated from the patient is in a phylogenetic cluster with three other study subjects (See Fig 1B, Cluster 7)
CY333	F	53	06/11	N/A	06/11	Cyprus	HC	539	0.05	Cypriot citizen. Infected in Cyprus. She was diagnosed HIV-1 positive after her spouse was found HIV-1 positive.
CY335	M	35	08/11	09/10	07/11	Cyprus	HBC	789	0.24	Cypriot citizen. Infected in Cyprus. Reported having sex with men.
CY336	M	25	09/11	07/05	08/11	Cyprus	HBC	N/A	9.26	Cypriot citizen. Infected in Cyprus. Homosexual partner of study subject CY337. Noted that HIV-1 strain isolated from this study subject is not phylogenetically related to HIV-1 strain isolated from CY337.
CY337	M	33	09/11	07/05	08/11	Cyprus	HBC	340	1.71	Cypriot citizen. Infected in Cyprus. Homosexual partner of study subject CY336. Diagnosed with syphilis in the past. HIV-1 strain isolated from the patient is in a phylogenetic cluster with three other study subjects (See Fig 1B, Cluster 7)
CY338	M	32	09/11	02/09	08/11	Cyprus	IDU	653	1.92	Cypriot citizen. Married to study subject CY 339. He reported injecting anabolic drugs using non-sterile syringes in Cyprus. HIV-1 strain isolated from the patient is in a phylogenetic cluster with study subject CY339 (See Fig 1B, Cluster 11).
CY339	F	39	09/11	08/08	08/11	Cyprus	HC	665	0.69	Cypriot citizen. Infected in Cyprus. Heterosexual partner of study subject CY338. HIV-1 strain isolated from the patient is in a phylogenetic cluster with study subject CY338 (See Fig 1B, Cluster 4)
CY340	F	31	09/11	07/05	09/11	Cameroon	OHPC	N/A	N/A	Cameroon citizen. She has a refugee status in Cyprus. Husband died in accident with unknown HIV-1 status.
CY341	M	37	09/11	02/11	08/11	Cyprus	HBC	330	4.55	Cypriot citizen. He reported that the likely route of HIV-1 infection was unprotected group sex in Cyprus. Diagnosed HIV-1 positive during hospitalization with fever, diarrhea, vomiting and wasting syndrome between in 2011.
CY342	M	40	09/11	N/A	08/11	Cyprus	HBC	279	2.57	Cypriot citizen. Infected in Cyprus. Diagnosed HIV-1 positive during blood donation. HIV-1 strain isolated from the patient is in a phylogenetic cluster with three other study subjects (See Fig 1B, Cluster 7)
CY343	M	50	09/11	06/05	07/11	Cyprus	HBC	509	28.70	Cypriot citizen. Infected in Cyprus. His wife is HIV-1 negative. Reported having sex with men. HIV-1 strain isolated from the patient is in a phylogenetic cluster with seven other study subjects (See Fig 1B, Cluster 12)
CY345	M	33	09/11	05/11	08/11	Cyprus	HBC	776	1.09	Infected in Cyprus. Reported having sex with men.
CY346	M	41	09/11	N/A	09/11	Greece	HBC	51	184.00	Greek citizen. Infected in Cyprus; Homosexual partner of CY347. It is likely to be HIV-1-infected since 2009 when he was hospitalized with fever and lymphedema, however HIV-1 status at that time is unknown. HIV-1 strain isolated from the patient is in a large phylogenetic cluster with eight other patients including study subject CY347 (See Fig 1B, Cluster 10)
CY347	M	27	09/11	N/A	09/11	Greece	HBC	237	2.17	Greek citizen. Arrived in Cyprus in 2009. Infected in Cyprus. HIV-1 strain isolated from the patient is in a large phylogenetic cluster with eight other patients including study subject CY346 (See Fig 1B, Cluster 10)
CY348	F	23	10/11	N/A	- /98	Romania	Vaccination	634	0.08	Romanian citizen. Infected in Romania. The most likely route of HIV-1 infection was a vaccination procedure in Romania.
CY349	M	36	10/11	N/A	N/A	Serbia	N/A	424	1.97	Serbia citizen. HIV-1 strain isolated from the patient is in a large phylogenetic cluster with eight other patients (See Fig 1B, Cluster 10)
CY350	M	28	12/11	N/A	11/11	Cyprus	HBC	861	1.96	Cypriot citizen. Infected in Cyprus. HIV-1 strain isolated from the patient is in a phylogenetic cluster with three other study subjects (See Fig 1B, Cluster 5)
CY351	M	27	12/11	N/A	11/11	Cyprus	HC	264	5.23	Cypriot citizen. Infected in Cyprus. Diagnosed HIV-1 positive during pre-surgery routine testing procedures.
CY352	M	30	01/12	N/A	10/06	Cyprus	HBC	922	0.17	Cypriot citizen. Infected in the U.S. in 2006. Returned in Cyprus in 2011.
CY353	M	37	01/12	01/11	11/11	Cyprus	HBC	761	33.60	Cypriot citizen. Infected in Greece. Reported having sex with men. Had a sexual relationship with an HIV-1 positive person in Greece.
CY354	M	30	01/12	09/10	12/11	Cyprus	HBC	555	4.35	Cypriot citizen. Infected in Cyprus. Reported having sex with men. HIV-1 strain isolated from the patient is in a phylogenetic cluster with seven other study subjects (See Fig 1B, Cluster 12).
CY355	M	30	01/12	03/11	01/12	Bulgaria	HBC	155	3.95	Bulgaria citizen. Infected in Cyprus. HIV-1 strain isolated from the patient is in a phylogenetic cluster with two other study subjects (See Fig 1B, Cluster 6)
CY356	M	28	02/12	N/A	07/05	Cyprus	HBC	386	4.96	Cypriot citizen. Infected in Greece in 2005.
CY357	M	52	02/12	N/A	01/12	Cyprus	HBC	168	14.00	Cypriot citizen. Infected in Cyprus. Diagnosed positive during hospitalization with pneumonia.
CY359	M	52	03/12	N/A	03/12	Ukraine	U	3	0.37	Ukraine citizen. Immigrated from Ukraine and resides in Cyprus since 2004. Diagnosed HIV-1 positive when hospitalized with pneumonia. Married with two daughters. Heterosexual partner of study subject CY362. HIV-1 strain isolated from the patient is in a phylogenetic cluster with CY362 (See Fig 1B, Cluster 3)
CY362	F	48	03/12	06/05	03/12	Ukraine	U	1,088	0.04	Ukraine citizen. Immigrated from Ukraine and resides in Cyprus since 2004. Heterosexual partner of study subject CY359. Infected in Cyprus. HIV-1 strain isolated from the patient is in a phylogenetic cluster with study subject CY359 (See Fig 1B, Cluster 3).
CY363	M	32	03/12	07/05	- /11	Cyprus	HBC	416	4.26	Cyprus citizen. Infected in Thailand. Reported having sex with men. HIV-1 strain isolated from the patient is in a phylogenetic cluster with study subject CY329 (See Fig 1B, Cluster 2)
CY364	M	38	03/12	07/05	03/12	Cyprus	HBC	234	59.20	Cyprus citizen. Infected in Cyprus. Diagnosed HIV-1 positive during blood donation testing procedures. HIV-1 strain isolated from the patient is in a large phylogenetic cluster with eight other study subjects (See Fig 1B, Cluster 10)
CY365	M	24	03/12	N/A	03/12	Cyprus	HBC	968	11.10	Cyprus citizen. Diagnosed HIV-1 positive during hospitalization with high fever and flu-like symptoms.
CY367	M	43	05/12	06/05	04/12	U.K.	HBC	264	1.41	U.K. citizen. Frequent international traveler. Diagnosed HIV-1 positive during an immigration entry visa application procedure.
CY368	M	33	05/12	09/11	N/A	Cyprus	HBC	489	0.87	Cyprus citizen. Infected in Cyprus. Diagnosed HIV-1 positive during routine blood analysis. HIV-1 strain isolated from the patient is in a phylogenetic cluster with seven other study subjects (See Fig 1B, Cluster 12)
CY369	M	29	06/12	06/05	02/12	Greece	HBC	487	1.77	Greek citizen. Infected in Greece. Genetic analysis of isolated HIV-1 strains revealed primary drug resistance mutation (K103N) for NNRTI.
CY370	M	47	06/12	07/05	N/A	Cyprus	HC	471	5.56	Cypriot citizen. HIV-1 strain isolated from the patient is in a large phylogenetic cluster with seven other study subjects (See Fig 1B, Cluster 12)
CY371	M	25	06/12	11/11	N/A	Cyprus	HC	427	2.42	Cypriot citizen. Infected in Cyprus. Found HIV-1 positive during a blood donation testing procedure. He reported having sex with many female partners.
CY372	M	47	06/12	N/A	N/A	Cyprus	HBC	707	0.22	Cypriot citizen. Reported having sex with men.
CY373	M	55	06/12	N/A	04/12	Cyprus	HC	432	1.06	Cypriot citizen. Infected in Cyprus. Diagnosed HIV-1 positive during routine blood analysis.
CY374	M	28	06/12	01/12	05/12	Cyprus	HBC	817	8.10	Cypriot citizen. He reported having unprotected sex with numerous male partners. HIV-1 strain isolated from the patient is in a large phylogenetic cluster with seven other study subjects (See Fig 1B, Cluster 12)
CY375	M	38	06/12	06/05	05/12	Bulgaria	HBC	412	1.38	Immigrated from Bulgaria since 2004. Infected in Cyprus. HIV-1 strain isolated from the patient is in a phylogenetic cluster with two other study subjects (See Fig 1B, Cluster 6)
CY376	M	27	07/12	04/11	N/A	Cyprus	HBC	670	13.90	Cypriot citizen. Infected in the U.K. Resides in the U.K. since 2006.
CY377	M	56	07/12	06/05	N/A	Cyprus	HC	582	3.30	Treated with Zovirax before his was diagnosed HIV-1 positive (date missing).
CY378	M	53	07/12	-/09	N/A	Cyprus	HC	582	34.50	Cypriot citizen. Diagnosed HIV-1 positive during hospitalization with high fever.
CY379	M	29	07/12	N/A	01/11	Cyprus	HBC	102	16.10	HIV-1 strain isolated from the patient is in a large phylogenetic cluster with eight other patients (See Fig 1B, Cluster 10). Signs of weight and muscle mass loss and physiological complications.
CY380	M	35	07/12	N/A	05/12	Kurdistan	HC	804	9.87	Kurdish refugee in Cyprus. Married to study subject CY381. Reported having an extramarital affair. Diagnosed HIV-1 positive during a pre-surgery diagnostic procedure. HIV-1 strain isolated from the patient is in a phylogenetic cluster with CY381 (See Fig 1B, Cluster 1)
CY381	F	37	07/12	N/A	- /12	Kurdistan	HC	319	12.60	Kurdish refugee in Cyprus. Wife of CY380. Diagnosed HIV-1 positive after her husband’s HIV-1 diagnosis. HIV-1 strain isolated from the patient is in a phylogenetic cluster with CY380 (See Fig 1B, Cluster 1)
CY382	M	26	07/12	03/12	06/12	Cyprus	HBC	1,191	0.18	Cypriot citizen. Infected in Cyprus. Reported having sex with men. Diagnosed HIV-1 positive on routine blood testing.
CY383	M	46	07/12	03/12	06/12	Cyprus	HBC	639	0.62	Cypriot citizen. Reported having sex with men.
CY384	M	74	07/12	03/11	06/12	Cyprus	HC	122	2.18	Cypriot citizen. Resided in the U.K. Found HIV-1 positive on routine pre-surgery blood testing.
CY385	M	31	08/12	N/A	N/A	Greece	HBC	1,102	< 55	Greek citizen who resides in Cyprus. Reported having sex with men. HIV-1 strain isolated from the patient is in a phylogenetic cluster with seven other study subjects (See Fig 1B, Cluster 12)
CY386	F	23	08/12	N/A	07/12	Romania	HC	90	0.319	Romanian citizen who lives in Cyprus. Infected in Cyprus. Reported having many heterosexual relationships. On HIV-1 CART since September 13, 2012 after hospitalization with pneumonia and CMV brain infection.
CY387	F	25	09/12	N/A	08/12	Mali	HC	295	7.94	Mali citizen. Immigrated from Mali. Most likely, she was not infected in Cyprus.
CY388	M	37	09/12	N/A	09/12	Cyprus	HC	745	1.44	Cypriot citizen. HIV-1 strain isolated from the patient is in a large phylogenetic cluster with seven other study subjects (See Fig 1B, Cluster 12), most of whom reported having sex with men.
CY389	M	32	09/12	05/12	N/A	Cameroon	U	381	1.27	Cameroon citizen. Immigrant from Cameroon. Most likely, he was not infected in Cyprus.

^a^ Indicates the laboratory code for each study subject as described in the text.

^b^ F, female; M, male.

^c^ Indicates the age of the study subject at the time of data collection; N/A, not available.

^d^ Indicates the date (month/year) of the sample collection.

^e^ Indicates the date (month/year); N/A, not available.

^f^ Indicates the date (month/year); N/A, not available.

^g^ Country of birth; U.K., United Kingdom; N/A, not available.

^h^ HC, heterosexual contact; OHPC, originating from high prevalence country; HBC homo/bisexual contact; IDU, Injecting Drug User; U, unknown; OE, occupational exposure; N/A, not available.

^I^ Information provided by the study subjects. CART, combined antiretroviral therapy; CMV, cytomegalovirus; N/A, not available.

The main characteristics of the study subjects are presented in Table 2. Seventy-eight individuals out of the one hundred participating individuals (78%) are male and the remaining twenty-two (22%) are female with the median age of 39 years (IQR, 33–47). Sixty-three subjects were Cypriot citizens living permanently in Cyprus at the time of study, even though a number of them reported traveling or living abroad in the past, whereas the remaining thirty-seven subjects were born abroad: Romania (twelve study subjects); Greece (five); Cameroon (five); United Kingdom (three); Ukraine (three); Bulgaria (two); Kurdistan (two); Mali (two); Togo (one); Lithuania (one) and Serbia (one). The most common reported risk factor of HIV-1 transmission was homo/bisexual contact (HBC) (51%) followed by heterosexual contact (HC) (33%), other risk factors (4%), unknown risk factor (6%) and not available data (6%). Investigation for other sexually transmitted diseases showed that three study subjects were infected by the herpes simplex virus (HSV), one patient with syphilis and two patients were diagnosed with anal warts (condyloma acuminatum) which are caused by the human papilloma virus (HHV). Furthermore, one study subject (1%) was infected with hepatitis B virus (HBV) and two others (2%) with hepatitis C virus (HCV). At the time of HIV-1 sero-diagnosis, the median CD4 count and the plasma virus load were 424 cells/μl (IQR, 223–653) and 4.41 log copies/ml (IQR, 4.09–4.91), respectively. Analyses of the HIV-1 *pol* sequences (nucleotide positions 2253 to 3359 of the HXB2 genome) indicated that subtypes B and A1 were the most common subtypes present and accounted for 41% and 19% respectively, followed by subtype F1 (8%), C (7%), CRF02_AG (4%), A2 (2%), other CRFs (7%) and unknown recombinant forms (URFs) (12%). The sequences obtained in this study were submitted to the Genbank and the accession numbers (*pol* sequences, KJ635931 –KJ636030) will be available prior to the publication of the manuscript.

**Table 2:** Characteristics of the study subjects.

*Characteristics*	*Patients (%)*
**Gender** (%)	
Female	22 (22)
Male	78 (78)
**Age** (years)	
median (IQR) ^*a*^	39 (33–47)
**Countries of origin** (%)	
Cyprus	63 (63)
Romania	12 (12)
Greece	5 (5)
Cameroon	5 (5)
United Kingdom	3 (3)
Ukraine	3 (3)
Bulgaria	2 (2)
Kurdistan	2 (2)
Mali	2 (2)
Togo	1 (1)
Lithuania	1 (1)
Serbia	1 (1)
**Route of transmission** (%)	
HC ^*b*^	33 (33)
HBC ^*c*^	51 (51)
Other	4 (4)
Unknown	6 (6)
Not available	6 (6)
**Plasma HIV-RNA** (log copies/ml)	
median (IQR)	4.41 (4.09–4.91)
**CD4 count** (cells/ml)	
median (IQR) ^*d*^	424 (223–653)
**Subtype** (%)	
A_1_	19 (19)
A_2_	2 (2)
B	41 (41)
C	7 (7)
F_1_	8 (8)
CRF02_AG	4 (4)
Other CRFs ^*e*^	7 (7)
URFs ^*f*^	12 (12)
**Coinfection** (%)	
HBV	1 (1)
HCV	2 (2)

^a^ IQR, Interquartile Range.

^b^ HC, Heterosexual.

^c^ HBC, Homo/bisexual.

^d^ Information available for 91 patients.

^e^ CRFs, Circulating Recombinant Forms.

^f^ URFs, Unique Recombinant Forms.

A summary of the characteristics of patients with transmitted drug resistance mutations is shown in Table 3. The overall prevalence of transmitted drug resistance mutations (TDRM) to current HIV-1 antiretroviral drugs among the newly-diagnosed and drug-naive patients of the study cohort was 3.7% (3 out 82 study subjects). All of them were infected with viruses carrying a single TDRM. Specifically, there was one patient (CY369) infected with an HIV-1 strain with a non-nucleoside reverse transcriptase inhibitor (NNRTI) resistance mutation (K103N) and two patients (CY274 and CY314) with a protease inhibitor (PI) resistance mutation (M46L). CY369 is a 29-year old man born in Greece, infected with a sub-subtype A1 HIV-1 strain within the reported HBC transmission risk group. CY274 is a 53-year-old male born in the United Kingdom, infected with a subtype B strain within the reported HC transmission risk group and CY314 is 47-year-old female born in Romania, infected with a sub-subtype A2 strain and unknown transmission group. Additionally, there were two chronically sub-subtype F1 HIV-1-infected individuals from Romania (>52 weeks since diagnosis) with multiple drug resistant mutations. Study subject CY289, a Romanian citizen living in Cyprus, was infected with a sub-subtype F1 dual-class RT resistant strain carrying both K219Q and K103N mutations. The second study subject, also a Romanian citizen living in Cyprus, was also infected with a sub-subtype F1strain dual-class RT and PI resistant strain carrying D67N, K70R, M184V, T215F, Y181C, M46L, I54V and V82F mutations associated with drug resistance. Both study subjects reported, however, that they were diagnosed HIV-1 seropositive in Romania prior to their arrival in Cyprus and both of them were receiving cART in Romania (Table 1). Therefore, these patients were not classified as drug naïve, and consequently, the identified resistance mutations associated with these patients were excluded from the transmission of HIV drug resistance analysis.

**Table 3:** Characteristics of patients with drug resistance mutations.

*Patient* ^*a*^	*Sex* ^*b*^	*Age (years)*	*Weeks since diagnosis* ^*c*^	*Country of origin* ^*d*^	*Transmission risk group* ^*e*^	*CD4 (cells/mm*^*3*^*)*	*Plasma HIV-1 RNA (copies x 10*^*4/*^*ml)*	*Subtype*	*Surveillance drug resistance mutations* ^*f*^		
									*NRTI*	*NNRTI*	*PI*
CY274	M	53	1	U.K.	HC/Tattoo	N/A	1.85	B	-	-	M46L
CY314	F	47	1	Romania	U	285	2.17	A2	-	-	M46L
CY369	M	29	5	Greece	HBC	487	1.77	A1	-	K103NNNN	-

^a^ Indicates the laboratory code for each study subject.

^b^ F, female; M, male.

^c^ Indicates the duration from the first known positive HIV-1 antibody test.

^d^ Country of birth of the study subjects; U.K., United Kingdom.

^e^ HBC, homo/bisexual contact; HC, heterosexual contact; U, Unknown; N/A, data not available

^f^ NRTI, nucleoside reverse transcriptase inhibitor; NNRTI, non-nucleoside reverse transcriptase inhibitor; PI, protease inhibitor.

**Fig 1:**
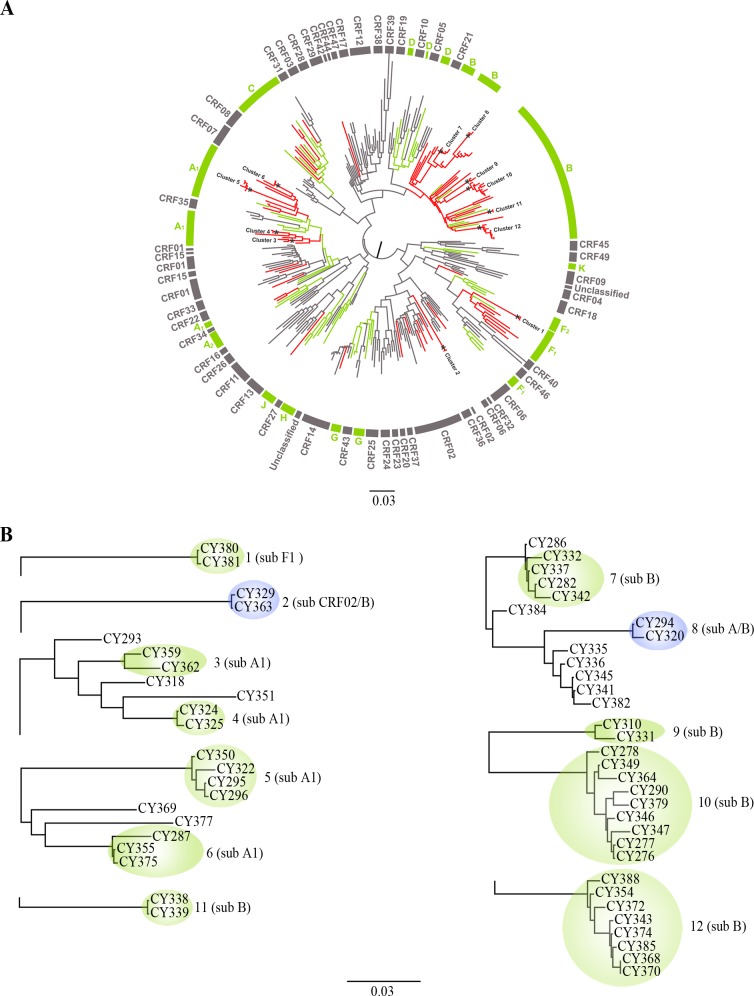
Phylogeny. **(A)** Maximum likelihood (ML) phylogenetic tree of *pol* (protease and partial reverse transcriptase) sequences from Cyprus (red) and a globally sampled reference dataset of sequences with known HIV-1 subtype. The circular brackets on the periphery of the tree indicate the HIV-1 subtype as follows: pure subtypes (green), circulating recombinant forms (CRFs) (grey) and unknown recombinant forms (URFs) (open bracket). Phylogenetic transmission clusters consisted of sequences from Cyprus are indicated with an asterisk (*) and numbered from 1 to 12. Scale indicates the number of nucleotide substitutions per site. **(B)** The twelve phylogenetic transmission clusters from Cyprus are magnified separately for the purpose of clarity.

Phylogenetic analyses, presented in the Fig 1A, discovered twelve phylogenetic transmission clusters highly supported by the set bootstrap support and the genetic distance criteria. Four of the clusters (Fig 1B: Cluster No3, No4, No5 and No6) included individuals infected with sub-subtype A1 strains, five clusters (Cluster No7, No9, No10, No11 and No12) with subtype B, one cluster with sub-subtype F1 (Cluster No1), one cluster with CRF02/B (Cluster No2) and one cluster with A/B recombinant strains (Cluster No8). The clinical and epidemiological information for all the individuals identified in the phylogenetic clusters are presented in Table 1.

Sub-subtype A1 clusters included: i) a heterosexual couple from Ukraine who were living in Cyprus since 1994 (No3), ii) a heterosexual couple from Greece (male) and Romania (female) who reported living in Cyprus since 1990 and 2007, respectively (No4), iii) four Cypriot men, all of whom are MSM (No5), and iv) three men, two from Bulgaria and one from Cyprus, all of whom are MSM. The two men from Bulgaria reported that HIV-1 infection was likely contracted in Cyprus (No6).

**Fig 2:**
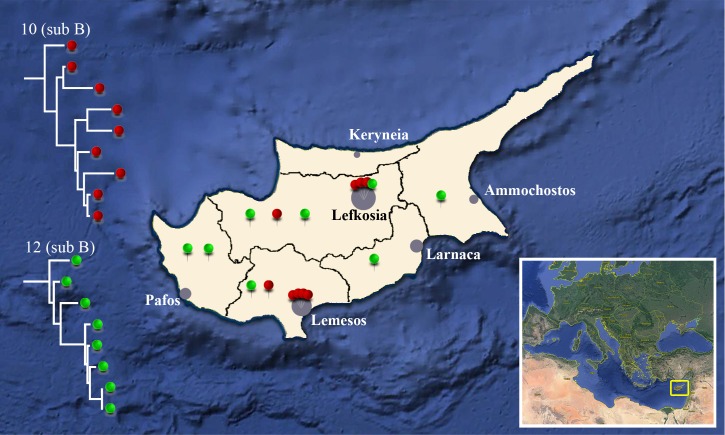
Phylogeography. Distribution of places of residence of patients of two large HIV-1 subtype B phylogenetic clusters in Cyprus (clusters 10 and 12). Partial phylogenetic trees for clusters 10 and 12 are shown on the left of the map of Cyprus. Inset map on the bottom right shows the position of Cyprus in Europe. Seventeen places of residence in total for the patients from cluster 10 (nine patients indicated in red) and 12 (eight patients indicated in green) are shown on six counties (Ammochostos, Keryneia, Lemesos, Larnaca, Lefkosia and Pafos). Black lines indicate the boundaries on the counties and gray-filled circle indicate the major city in each county.

Subtype B cluster No7 involved four men from Cyprus, all of them reported MSM. One of them reported HIV-1 contraction was likely contracted in Thailand or Russia by unprotected sex with a transsexual person. The other three men involved in this cluster reported that HIV-1 was likely contracted in Cyprus, two of them by unprotected sex with anonymous person and one of them with his partner who is not a study subject. Subtype B cluster No9 involved two men from Cyprus. One of them was a divorced thirty-year-old man who reported that HIV-1 infection was likely contracted in Greece by unprotected heterosexual contact, whereas the other one was a thirty-five-year-old married man who reported that HIV-1 infection was likely contracted in Cyprus by unprotected heterosexual contact with an anonymous person. The largest subtype B cluster, No10, involved nine men (six from Cyprus, one from Greece and one from Serbia) all of whom were MSM. Five of them reported that HIV-1 was contracted in Cyprus and one in Syria. Among the study subjects found in this cluster, there were two reported homosexual couples. The first couple consists of the study subjects CY276 and CY277, and the second one of CY346 and CY347. Furthermore, it was reported that the study subject CY349 is the homosexual partner of CY267, who was included in a previous molecular epidemiology study [11]. The median age of the study subjects found within this cluster is 32 years. Additionally, it was reported that four of them were living in the city of Limassol and three in Nicosia, whereas only two subjects were living in the rural areas of Nicosia and Limassol (one in each area). Subtype B cluster No12 is the second largest cluster identified in this study and it is consisted of eight men (seven from Cyprus and one from Greece). Six of them reported that the most likely route of infection was a hetero-bisexual contact, whereas two of them a heterosexual contact. Six of them reported that HIV-1 was contracted in Cyprus. The median age of the study subjects found within this cluster is 38 years. It was also reported that seven of them were living in rural areas of Lefkosia (two), Larnaca (one), Lemesos (one), Pafos (two) and Ammochostos (one), whereas only one subject was living in the city of Lefkosia. The geographical distribution of study subjects involved in clusters No10 and No12 are shown in Fig 2. Subtype B cluster No11 consisted of a heterosexual married couple (study subjects CY338 and CY339) from Cyprus. The wife (CY339) reported that she contracted HIV-1 infection in Cyprus by unprotected heterosexual contact with her husband (CY338).

Sub-subtype F1 cluster No1 involved a heterosexual married couple from Kurdistan, CRF02/B cluster No2 consisted of two Cypriot men (one of them reported himself as MSM), and A/B recombinant cluster No8 consisted of two men, one from Romania and one from Cyprus.

## Discussion

In this prospective study we are presenting the molecular epidemiology of HIV-1 infection and TDR in newly diagnosed patients in Cyprus between 2010 and 2012. Eighty-two newly diagnosed untreated patients participated in this study by providing demographic, epidemiological and behaviour information. This number represents the 53.6% of the antiretroviral naïve newly diagnosed patients, reported at the AIDS Clinic of the Larnaca General Hospital in Cyprus the period 2009–2010. The subjects were predominantly young Cypriot men reporting having sex with other men (MSM). The non-Cypriot subjects were mostly young people from Eastern European and African countries.

Previous molecular epidemiology studies conducted in Cyprus from 1986 to 2009 have shown that the predominant subtypes were subtypes A, B, C and CRF02_AG [11–13]. Our study reveals that subtypes B and A1 were the most common subtypes in Cyprus and accounted for 41.0 and 19.0% respectively, followed by subtype C (7.0%), F1 (8.0%), CRF02_AG (4.0%), A2 (2.0%), other CRFs (7.0%) and URFs (12%). Significantly, twenty-eight out of forty-one MSM study subjects (68.0%) were implicated in five transmission clusters, two of which were consisted of sub-subtype A1 and three of subtype B strains. The two largest MSM subtype B clusters included nine and eight Cypriot men, respectively, living in all major cities and rural areas in Cyprus (area controlled by the Republic of Cyprus). Furthermore, our findings indicate that within the study cohort of newly diagnosed drug naïve subjects, the prevalence of TDR to current HIV-1 antiretroviral drugs was only 3.66%, which is significantly lower than known prevalence rates of TDR in newly diagnosed patients in other European countries [5].

In conclusion, the results of the present study indicate that the HIV-1 infection in Cyprus is increasingly identified among young Cypriot MSM being infected in Cyprus. By comparing the results of previous HIV-1 transmission studies in Cyprus conducted between 1986 and 2009, which showed that new HIV-1 infections were more frequently detected among immigrants mostly from African countries, the current results point to a changing HIV-1 transmission dynamic in the island. Also, the TDR remains consistently low (4%) in Cyprus in comparison to Europe where it is stable at around 8% [5].

## Sequence data

GeneBank accession numbers for the sequences obtained in this study are as follows: *pol* sequences, KJ635931 –KJ636030.
